# Evidence for dopamine production and distribution of dopamine D_2_ receptors in the equine gastrointestinal mucosa and pancreas

**DOI:** 10.1371/journal.pone.0298660

**Published:** 2024-02-27

**Authors:** Nicolas C. Galinelli, Nicholas J. Bamford, Melody A. de Laat, Martin N. Sillence, Patricia A. Harris, Simon R. Bailey

**Affiliations:** 1 Melbourne Veterinary School, Faculty of Science, The University of Melbourne, Parkville, Victoria, Australia; 2 School of Biology and Environmental Science, Faculty of Science, Queensland University of Technology, Brisbane, Queensland, Australia; 3 Equine Studies Group, Waltham Petcare Science Institute, Melton Mowbray, United Kingdom; Zhejiang University of Technology, CHINA

## Abstract

Insulin dysregulation in horses is characterised by hyperinsulinaemia and/or tissue insulin resistance and is associated with increased risk of laminitis. There is growing evidence in other species that dopamine attenuates insulin release from the pancreas; however, this has yet to be examined in horses. The present study aimed to identify whether there are cells capable of producing or responding to dopamine within the equine gastrointestinal mucosa and pancreas. Tissue samples were collected from the stomach, small and large intestines, and pancreas of six mature horses following euthanasia. Samples of stomach contents and faeces were also collected. Immunohistochemistry was performed to identify tyrosine hydroxylase (TH), the rate-limiting enzyme for dopamine production, and dopamine D_2_ receptors in tissue sections. Additional immunostaining for glucagon, insulin and chromogranin A was performed to identify α cells, β cells and enteroendocrine cells, respectively. Gastric parietal cells expressed both TH and D_2_ receptors, indicating that they are capable of both producing and responding to dopamine. Dopamine was quantified in stomach contents and faeces by high-performance liquid chromatography with electrochemical detection, with similar concentrations found at both sites. Dopamine D_2_ receptors were expressed in duodenal epithelial cells but not more distally. A subset of enteroendocrine cells, located sporadically along the gastrointestinal tract, were found to be immunopositive for the D_2_ receptor. In pancreatic islets, TH was present in α cells, while D_2_ receptors were strongly expressed in β cells and variably expressed in α cells. These findings are consistent with studies of other species; however, dynamic studies are required to further elucidate the role of dopamine in the modulation of insulin and glucagon secretion in horses. This descriptive study provides preliminary evidence for a potential role of dopamine to act as a paracrine messenger in the gastrointestinal mucosa and endocrine pancreas of horses.

## Introduction

Insulin dysregulation (ID), encompassing hyperinsulinaemia and/or tissue insulin resistance, is a central feature of equine metabolic syndrome (EMS) and is associated with an increased risk of laminitis in horses [1, 2]. There is a growing body of evidence in other species that dopamine (produced locally, centrally or from the gastrointestinal tract) plays an important role in the attenuation of glucose-stimulated insulin secretion from the pancreas [3–5]. Whether dopamine exerts a similar physiological effect in horses is unclear. There is evidence that bromocriptine, a dopamine receptor agonist, can reduce the postprandial insulin response in horses with and without ID [6], while a postprandial rise in plasma L-DOPA (a precursor of dopamine) has been demonstrated during oral glucose tests in horses [7], with speculation that a lack of inhibition of insulin secretion by dopamine could be linked to ID [7]. Therefore, there is interest in investigating the potential role of dopamine in the pathogenesis and treatment of ID in horses.

Dopamine is a catecholamine produced by neurons of the central and peripheral nervous systems, as well as in other tissues. Outside of roles as a neurotransmitter and as a precursor for the formation of other catecholamines, dopamine acts within the gastrointestinal tract as a paracrine messenger to reduce gastrointestinal motility [8], inhibit gastric acid secretion [9] and increase duodenal bicarbonate secretion, with evidence that dopamine is secreted into the gastric lumen by parietal cells [10]. The secretion of insulin (by β cells) and glucagon (by α cells) from pancreatic islets is subject to negative control by dopamine in humans and rodents. Dopamine production within pancreatic islets themselves could represent an important paracrine regulatory mechanism for the secretion of insulin and glucagon [3, 11]. In horses, these physiological processes are yet to be characterised.

The rate-limiting enzyme for dopamine production is tyrosine hydroxylase (TH), and dopamine exerts its effects through several receptor subtypes, classified as D_1_ to D_5_. The D_2_ receptor subtype is an important regulator of metabolism and has been widely investigated in studies of both the pancreas and gastrointestinal tract in humans and rodents [12, 13]. The objectives of the current study were: (1) to identify whether there are cells capable of producing or responding to dopamine (through the expression of TH or D_2_ receptors, respectively) within equine gastrointestinal mucosa, (2) to measure dopamine concentrations in stomach contents (compared with faeces) of horses, and (3) to characterise the expression and distribution of TH and D_2_ receptors within the equine pancreas.

## Materials and methods

### Animals and tissue collection

Tissue samples were collected from six adult horses following euthanasia. Horses were donated to the Melbourne Veterinary School, with signed owner consent obtained for each horse, including authorisation to conduct post mortem examinations for teaching purposes and to collect tissue specimens for research studies. Horses were euthanised on humane grounds by the referring veterinarian and none were euthanised exclusively for teaching or research purposes. The inclusion criteria comprised adult (but not aged or geriatric) horses that were in good body condition and declared by the referring veterinarian to have no history of gastrointestinal or endocrine disorders, as well as no macroscopic evidence of gastrointestinal or endocrine diseases on post-mortem examination. Breeds of horses included Thoroughbred (*n* = 4), Standardbred (*n* = 1) and Thoroughbred-cross (*n* = 1), with a median age of 4.5 years (range, 2.5 to 11 years; S1 Table). Reasons for euthanasia included cervical stenotic myopathy (*n* = 3), large parotid melanomas (*n* = 1), proximal sesamoid bone fracture (*n* = 1) and navicular bone necrosis (*n* = 1).

Samples were collected as soon as possible after euthanasia: within 2 to 6 hours for pancreatic samples and within 12 hours for gastrointestinal samples, provided the cadaver had been stored at 4°C. Full-thickness samples were collected from the fundic and pyloric gland regions of the stomach, as well as the pancreas, duodenum, jejunum, ileum, caecum, right ventral colon, left dorsal colon and descending colon. Tissues were placed in 10% buffered neutral formalin for 24 hours. Samples were then embedded in paraffin, and 3 μm thick sections were mounted on glass slides.

Samples of stomach contents were obtained from the six horses, along with samples of fresh faeces collected directly from the small colon. Samples were placed into sterile tubes (BD, Macquarie Park, NSW, Australia) and frozen at –80°C until further processing.

### Immunohistochemistry

Each section was deparaffinised in xylene (10 minutes), followed by stepwise immersion for 5 minutes in each of 100%, 95% and 80% ethanol, before being rinsed in distilled water. Antigen retrieval was performed using a 10 mM sodium citrate buffer solution, heated to 95°C for 30 minutes and allowed to cool for 20 minutes before rinsing with a washing buffer (PBS with 0.01% Tween 20). Sections were then blocked with 1% hydrogen peroxide for 5 minutes, followed by 20 minutes with bovine serum albumin solution (4% in PBS).

The primary antibodies used were both rabbit polyclonal antibodies; one against the D_2_ receptor (ab150532; Abcam, Melbourne, Victoria, Australia) and the other against TH (Gene Tex Inc., California, USA). These antibodies were applied at 1:1000 dilution (optimal dilution determined from pilot studies) in PBS with 1% bovine serum albumin, and slides were incubated overnight at 4°C in a humid environment. Following incubation, the slides were washed and incubated with the secondary antibody (polyclonal goat anti-rabbit IgG-HRP, ab6721; Abcam, Melbourne, Victoria, Australia) for 2 hours at room temperature. Diaminobenzidine substrate-chromogen solution (DAB substrate kit, ab64238; Abcam, Melbourne, Victoria, Australia) was used to develop the immunostaining and Mayer’s hematoxylin solution (ab220365; Abcam, Melbourne, Victoria, Australia) was applied as a counterstain. Sections were then dehydrated using ethanol (95% and 100%) and xylene immersion, and subsequently mounted using DPX mounting solution (Sigma-Aldrich, Sydney, NSW, Australia).

A section of equine adrenal gland was included as a positive control, with additional positive control slides including the submucosal (Meissner) and myenteric (Auerbach) plexus in gastrointestinal samples, which are known to contain dopaminergic neurons [14, 15]. Negative control slides were incubated in the absence of a primary antibody but otherwise processed using the same protocol. Standard hematoxylin and eosin staining was also performed to assist with cell identification.

To confirm the distribution of α and β cells in pancreatic islets, an anti-glucagon antibody (ab92517; Abcam, Melbourne, Victoria, Australia) and an anti-insulin antibody (Cell Signaling Technology, Danvers, Massachusetts, USA) were used, following the same protocol outlined above. To identify enteroendocrine cells in the gastrointestinal mucosa, an anti-chromogranin A antibody (ab220365; Abcam, Melbourne, Vic, Australia) was used [16, 17]. To evaluate tissues for colocalisation of antigens, consecutive sections (3 μm thickness) were stained with the relevant antibodies of interest, as previously reported [18].

Each slide was reviewed using light microscopy at two magnifications, 400x and 1000x (oil immersion), by two evaluators. The morphology and distribution of immunopositive cells were recorded for each tissue from each horse, and the relevant cell types identified [19].

### Dopamine quantification

Dopamine quantification in stomach contents and faeces was performed using high-performance liquid chromatography (515 HPLC pump, Waters Corporation, Milford, MA, USA) with electrochemical detection (Waters 464 ECD; Waters Corporation, Milford, MA, USA), as previously described for samples from the digestive tract of small rodents [20, 21]. Each sample was prepared following the instructions of a commercial kit (Chromsystems Instruments & Chemicals GmbH, Munich, Germany) for the measurement of catecholamines, which included extraction cartridges, buffers, standards, mobile phase solvent and column. Peaks were analysed using AcqKnowledge software (Biopac Systems Inc. Santa Barbara, CA, United States). Dopamine concentrations were calculated based on the ratio of the peak area to the internal standard (3,4 dihydroxybenzylamine), compared with calibration standards. The limit of detection (calculated as the lowest concentration to give a peak area >3 times greater than the background noise) was 8.3 pg/ml.

### Statistical analysis

Descriptive data were reported as median (range). Dopamine concentrations in stomach contents and faeces were compared using the Wilcoxon matched-pairs signed rank test. Data were analysed using GraphPad Prism software (version 9.1.2, GraphPad Software, La Jolla, CA, USA). Significance was accepted at *P* ≤ 0.05.

## Results

Unless specifically described, all horses demonstrated consistent immunostaining patterns across all sections of the gastrointestinal mucosa and pancreas evaluated.

### Immunohistochemistry of the gastrointestinal mucosa

**Fig 1:**
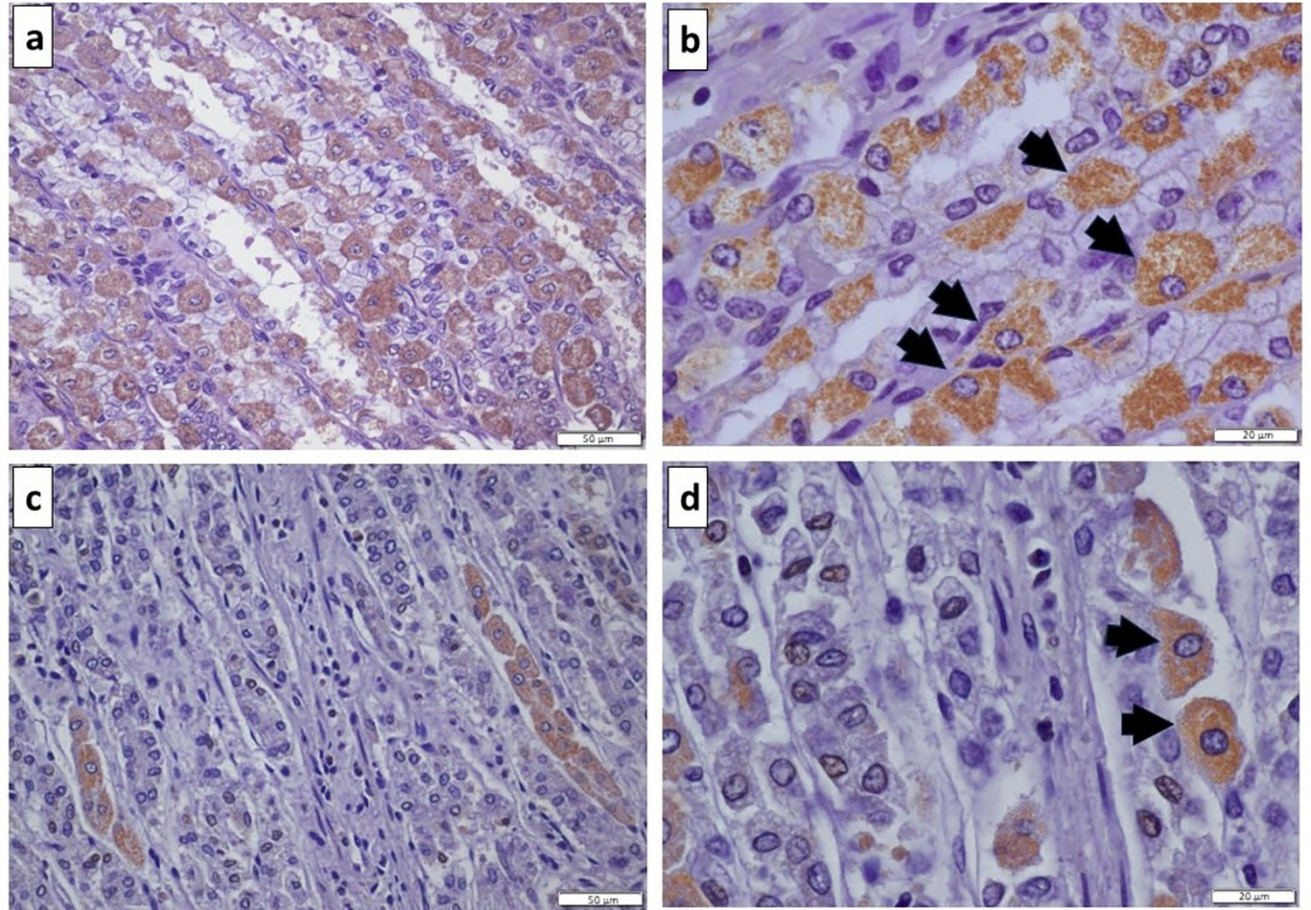
Tyrosine hydroxylase immunostaining of equine gastric mucosa. Fundic gland region at (a) lower magnification showing numerous immunopositive parietal cells (brown staining) within gastric pits, and (b) higher magnification with parietal cells indicated by black arrows. Pyloric gland region at (c) lower magnification showing relatively fewer immunopositive parietal cells (brown staining) within gastric pits, and (d) higher magnification with parietal cells indicated by black arrows.

In the stomach, immunostaining for TH was evident in parietal cells, which were readily identified by their characteristic large pyramidal morphology and eosinophilic appearance when stained with hematoxylin and eosin [19]. Parietal cells were numerous in the fundic gland region and relatively less abundant in the pyloric gland region, as expected (Fig 1). There were occasional surface epithelial cells that were immunopositive for TH in both the fundic and pyloric gland regions of the stomach. Dopamine D_2_ receptors were present on occasional cells located towards the base of gastric pits. These cells were generally pyramidal or flask-shaped, with a basal nucleus, and located between two epithelial cells, which were subsequently confirmed to be enteroendocrine cells by chromogranin A immunostaining.

**Fig 2:**
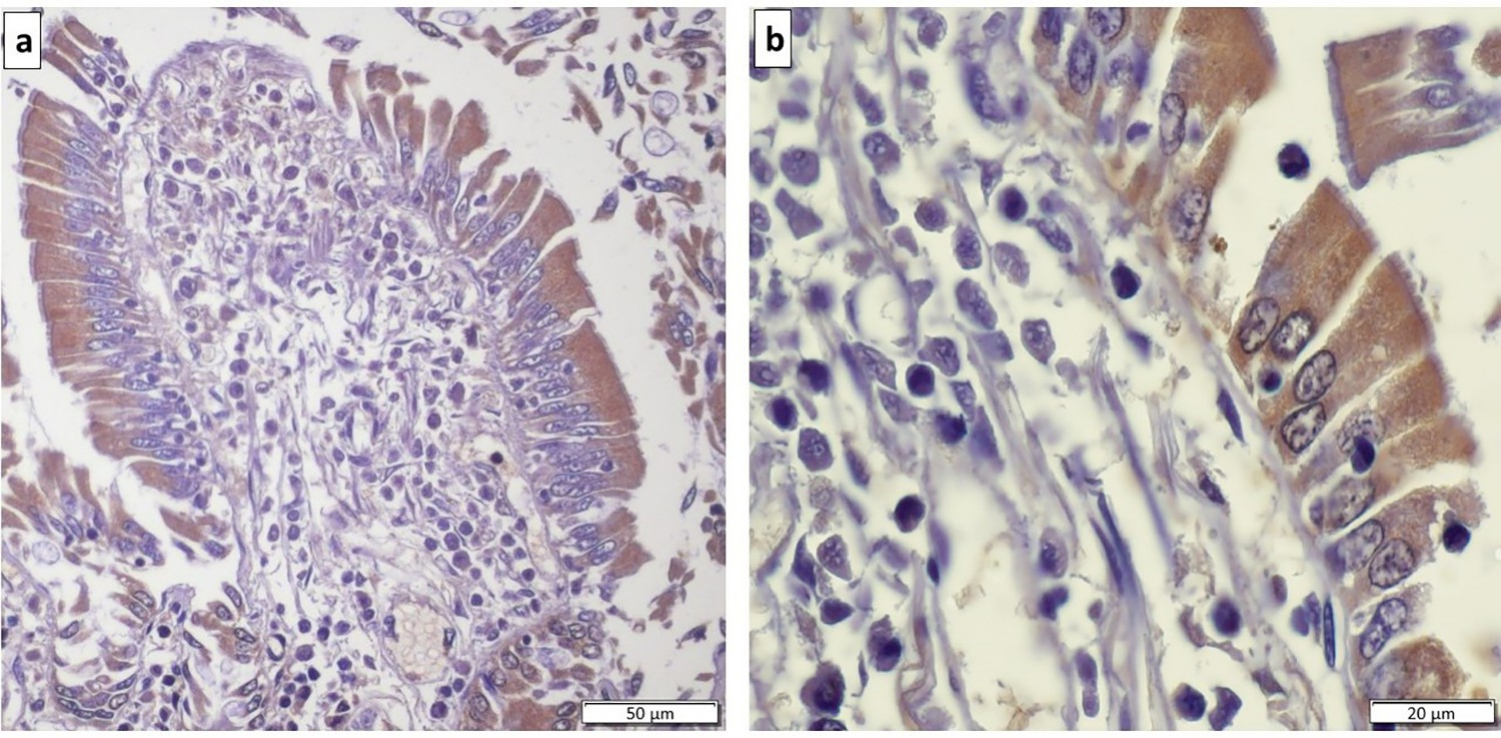
Dopamine D_2_ receptor immunostaining of equine duodenal mucosa. Duodenal epithelial cells, at (a) lower magnification and (b) higher magnification, demonstrating immunopositivity (brown staining) for D_2_ receptors.

**Fig 3:**
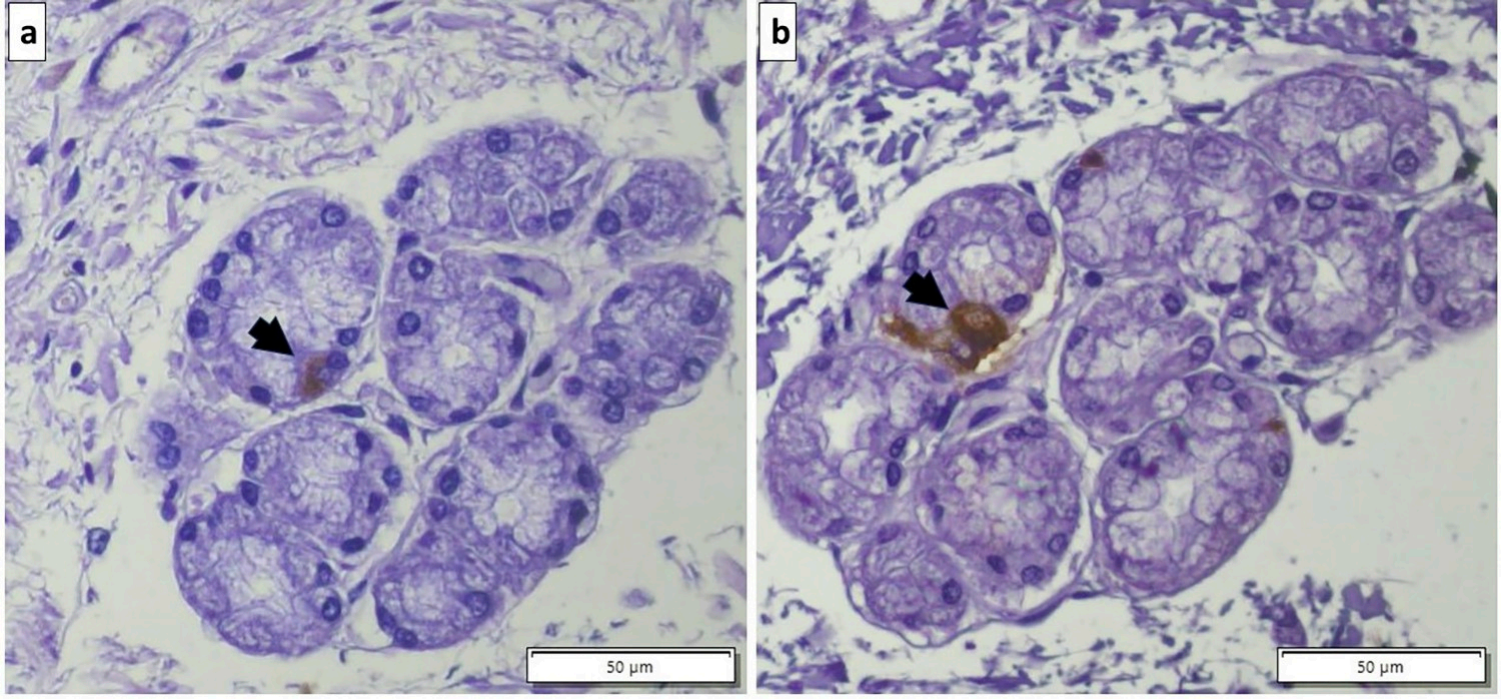
Dopamine D_2_ receptor and chromogranin A immunostaining of equine duodenum. Consecutive sections through a Brunner’s gland showing (a) a cell that is immunopositive for D_2_ receptors (black arrow), and (b) the same cell that is immunopositive for chromogranin A (black arrow), confirming this to be an enteroendocrine cell.

**Fig 4:**
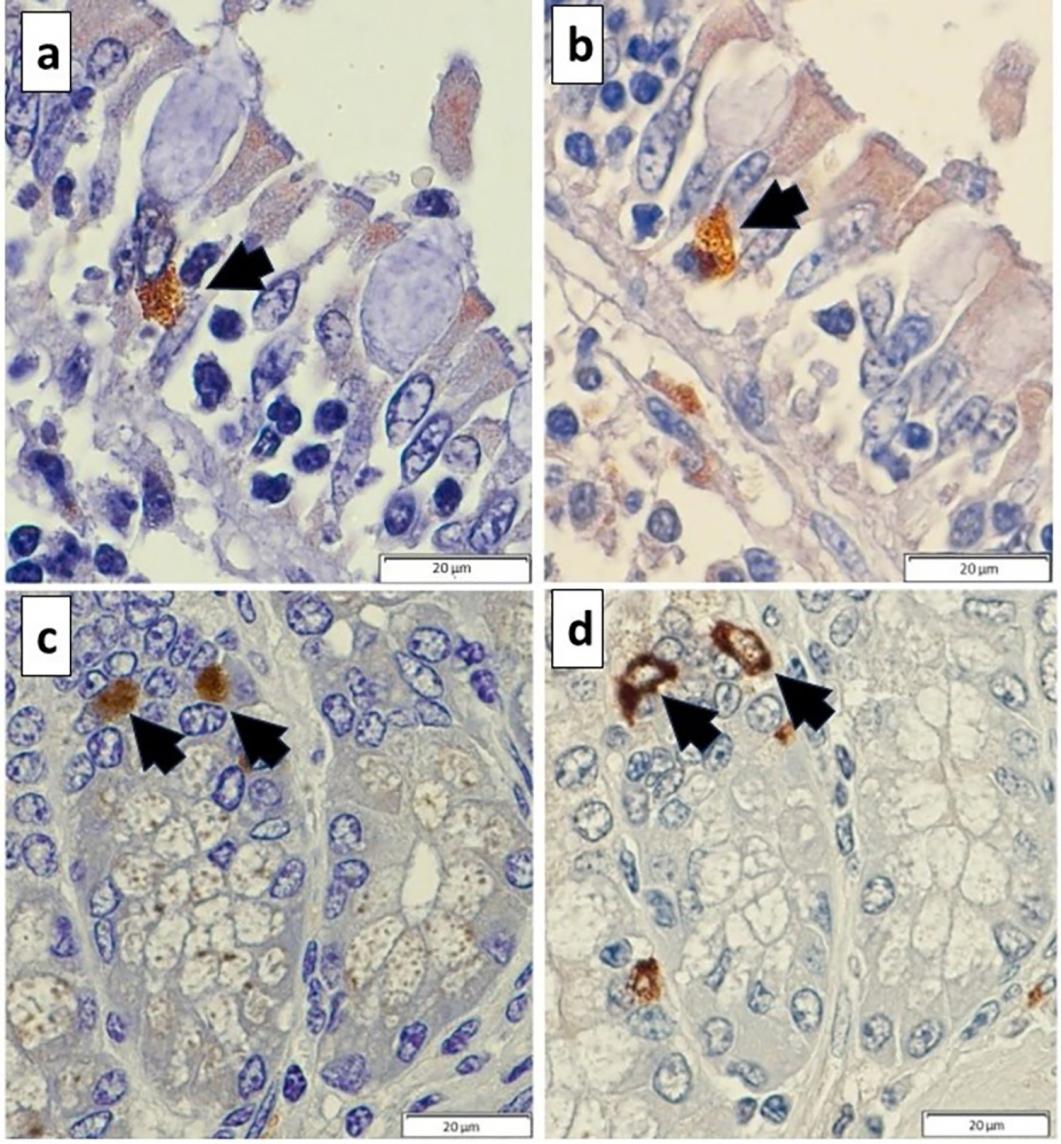
Dopamine D_2_ receptor and chromogranin A immunostaining of equine ileum. Consecutive sections through ileal mucosa showing (a) a cell located among surface epithelial cells that is immunopositive for D_2_ receptors (black arrow), and (b) the same cell that is immunopositive for chromogranin A (black arrow), confirming this to be an enteroendocrine cell. Consecutive sections through the ileal mucosa showing (c) cells located within intestinal crypts that are immunopositive for D_2_ receptors (black arrows), and (d) the same cells that are immunopositive for chromogranin A (black arrows), confirming these to be enteroendocrine cells.

In all sections of small and large intestine evaluated, TH was not detected in epithelial cells, but was found within the myenteric and submucosal plexus, and in some lymphoid cells, where present. In the duodenum, D_2_ receptors were consistently present on epithelial cells (Fig 2). In addition, occasional cells were immunopositive for D_2_ receptors within Brunner’s glands and in cells located between Paneth cells in the intestinal crypts, which were confirmed to be enteroendocrine cells by chromogranin A immunostaining (Fig 3). Dopamine D_2_ receptors were also identified on enteroendocrine cells in the surface epithelium and within intestinal crypts along the jejunum and ileum (Fig 4). Approximately 2 to 4 immunopositive cells per 400x field of view were noted in the mucosa throughout the small intestine and were less frequently encountered in the large intestine.

### Immunohistochemistry of the pancreas

**Fig 5:**
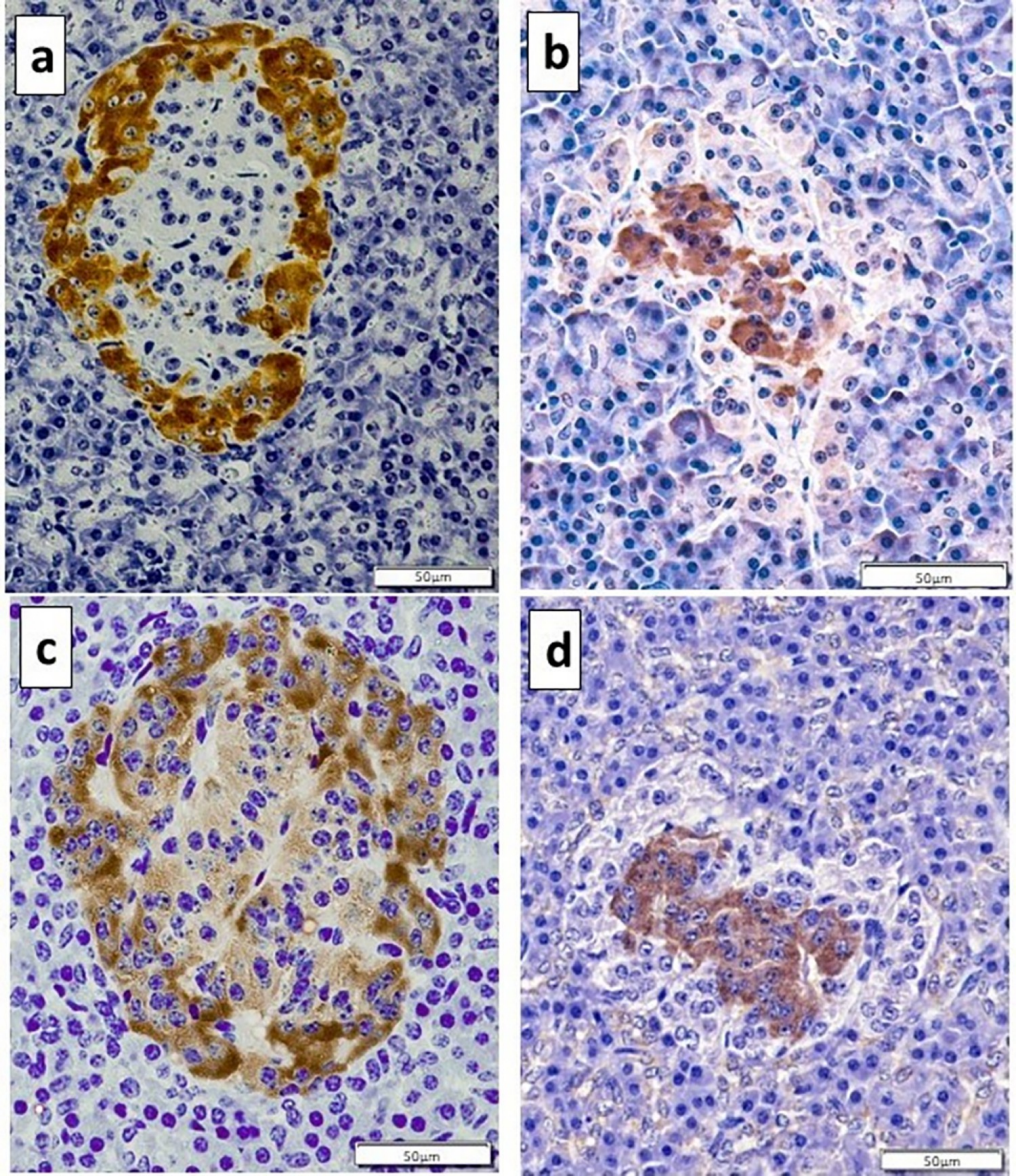
Immunostaining of equine pancreatic islets. (a): Immunostaining for insulin, identifying β cells around the periphery of the islet. (b): Immunostaining for glucagon, identifying α cells within the centre of the islet. (c) Immunostaining for the dopamine D_2_ receptor, demonstrating strong stain uptake among β cells with mild stain uptake among α cells. (d) Immunostaining for tyrosine hydroxylase, demonstrating stain uptake among α cells.

Immunostaining for insulin was evident within cells located at the periphery of pancreatic islets, confirming these to be β cells (Fig 5A). In contrast, immunostaining for glucagon was evident within cells located in the centre of pancreatic islets, confirming these to be α cells (Fig 5B). Immunostaining for D_2_ receptors occurred mainly at the periphery of pancreatic islets, consistent with these receptors being located on β cells, with variable immunostaining observed in centrally located cells (Fig 5C). Immunostaining for TH was predominantly confined to the central cells, consistent with this enzyme being expressed by α cells (Fig 5D). Exocrine regions of the pancreas did not express TH or the D_2_ receptor.

### Quantification of dopamine in stomach contents and faeces

**Fig 6:**
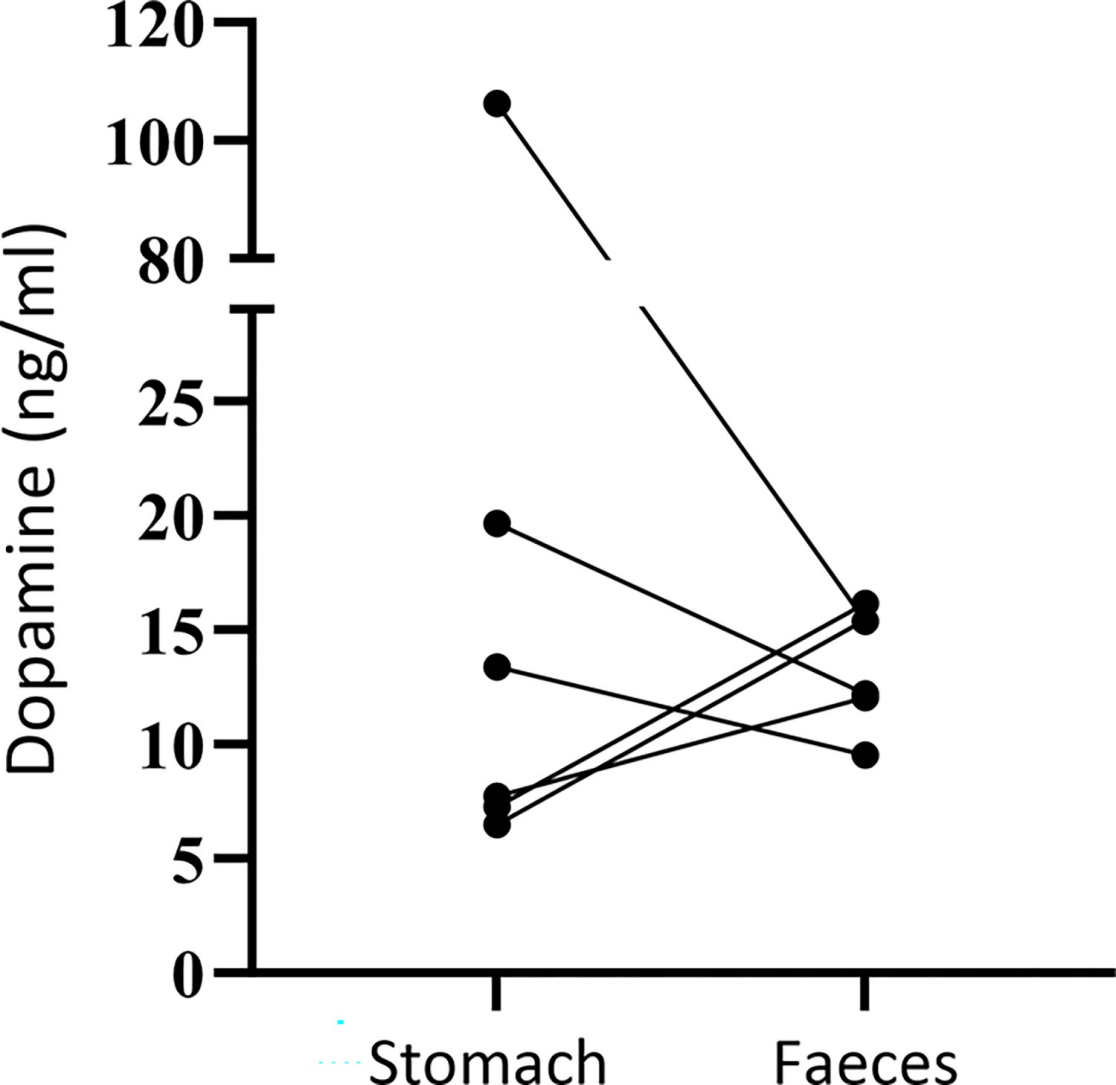
Dopamine concentrations in stomach contents and faeces of horses. Results determined by high-performance liquid chromatography with electrochemical detection. Lines join results from the same horse (*n* = 6). Dopamine concentrations were not significantly different between sites (*P* > 0.9, Wilcoxon matched pairs signed rank text).

Median dopamine concentration in stomach contents was 10.5 (range, 6.5–106.3) ng/ml and in faeces was 13.8 (range, 9.5–16.2) ng/ml (*P* > 0.9; Fig 6).

## Discussion

This study identified cells that are capable of producing dopamine (by expressing TH) and cells that are capable of responding to dopamine (by expressing D_2_ receptors) within the gastrointestinal mucosa and pancreatic islets of horses. Gastric parietal cells expressed both TH and D_2_ receptors, and enteroendocrine cells in the stomach and intestines were found to express D_2_ receptors. Within pancreatic islets, β cells strongly expressed D_2_ receptors, while α cells expressed TH with variable expression of D_2_ receptors. Dopamine was quantified in stomach contents and faeces, with similar concentrations found at both sites. These findings are relevant to studies of equine gastroenterology and endocrinology and indicate that further investigation of the role of dopamine as a paracrine messenger in the equine gastrointestinal tract and endocrine pancreas is warranted.

Studies of humans and rodents have confirmed that dopamine is secreted into the gastric lumen by parietal cells [22–24]. The actions of dopamine to reduce gastric motility [8], inhibit gastric acid secretion [9], and increase duodenal bicarbonate secretion [10, 25], have led to the conclusion that dopamine might play an important role in the protection against gastroduodenal ulceration [26]. These physiological actions of dopamine have not been characterised in horses. The present study confirmed that equine gastric parietal cells express TH and D_2_ receptors, meaning that they are capable of both producing and responding to dopamine. The duodenal epithelium is a key target for dopamine secreted into the stomach in other species [24, 27], with increased duodenal bicarbonate secretion following stimulation by dopamine [10]. The expression of D_2_ receptors on duodenal epithelial cells in the present study suggests that a similar physiological mechanism could also occur in horses. While further studies are required to investigate the dynamics of dopamine secretion by equine gastric parietal cells and the response of different cell types to dopamine binding, this study provides preliminary evidence for a role of dopamine as a gastrointestinal paracrine messenger in horses.

Dopamine was detected in stomach contents from all horses using high-performance liquid chromatography, providing evidence of dopamine production in the equine stomach. We expected to find higher concentrations of dopamine in stomach contents compared to faeces, but levels were similar at both sites. The presence of dopamine and other catecholamines in the large intestine of other species has been reported [21, 22], and microbial production of catecholamines is known to occur [20, 28]. Further studies to characterise the sites of dopamine production in the equine gastrointestinal tract are warranted.

Enteroendocrine cells are specialised epithelial cells that are dispersed along the gastrointestinal tract, producing a range of hormones that are involved in a variety of physiological processes [29]. Enteroendocrine cells can be classified as different cell types according to their specific hormone products, and their response to different stimuli, with evidence that certain enteroendocrine cells respond to dopamine [30]. The distribution of enteroendocrine cells in this study was consistent with previous reports in horses and in other species [16, 31]. We were not able to differentiate between individual cell types, as immunostaining for chromogranin A only enables the broad identification of an enteroendocrine cell [17]. The presence of D_2_ receptors on enteroendocrine cells suggests a potential role for dopamine to modulate the function of these cells, although further studies are certainly required to investigate this hypothesis.

There is interest in the physiological effects of dopamine on the endocrine pancreas due to evidence in people that dopamine receptor antagonists can cause hyperinsulinemia and glucose intolerance [32, 33], while conversely, dopamine agonists can reduce insulin secretion and improve glycemic control [34, 35]. In horses, the effect of dopamine on the endocrine pancreas and any potential link with ID, has not yet been characterised. There is evidence that bromocriptine, a dopamine receptor agonist, can reduce the postprandial insulin response in horses with and without ID [6]. However, this particular drug could also be acting through effects on α_2A_ receptors [36]. A metabogenomic study of Arabian horses identified carbidopa as a putative metabolite that was associated with phenotypic markers of EMS, including the modified insulin-to-glucose ratio as a marker of ID, although the significance of this finding remains unclear [37]. A postprandial rise in plasma L-DOPA has been demonstrated during oral glucose tests in horses, with the investigators of that study speculating that a lack of inhibition of insulin secretion by dopamine could be linked to ID [7].

In the present study, the distribution of β cells at the periphery and α cells in the centre of pancreatic islets was consistent with previous immunohistochemical studies of equine pancreatic islets [38, 39]. Immunostaining for TH was present in α cells, while D_2_ receptors were strongly expressed in β cells and variably expressed in α cells. In humans and rodents, both β cells and α cells are known to express D_2_ receptors, and α cells are capable of producing dopamine, which is proposed to exert a local paracrine effect within pancreatic islets [3, 12, 40, 41]. Despite significant improvements in understanding, the role of dopamine in regulating the secretion of insulin and glucagon from the endocrine pancreas is complex and remains to be fully understood [40, 42]. Despite the demonstration of dopamine D_2_ receptors and TH within pancreatic islets of horses, dynamic *in vitro* and *in vivo* studies are required to confirm the potential physiological effects of dopamine on the equine pancreas.

The variability of staining for D_2_ receptors in α cells, both within individual horses and between different horses, could be associated with metabolic status; however, that cannot be determined based on this study. Other factors that might influence immunostaining intensity could include autolytic processes, influenced by the time between euthanasia and sampling, internal body temperature or bacterial transmigration. Staining of consecutive thin sections with different antibodies was used to confirm colocalisation of antigens within cells (an accepted technique) [43], to ensure that TH and D_2_ receptors were being accurately assigned to β cells or α cells within islets. In addition, δ cells are a third cell type that are evenly distributed among β cells and α cells within equine pancreatic islets [39]; however, δ cells were not identified in this study and the expression of TH and D_2_ receptors in these cells was not determined.

There are several important limitations to highlight. The number of horses studied was small, only Thoroughbred and Standardbred horses were included, and there was a wide range of ages (although none would be considered geriatric). Therefore, the generalisability of findings to equids of other breeds or ages remains to be determined. Ante mortem endocrine testing to diagnose ID or pituitary pars intermedia dysfunction was not performed, and while horses did not demonstrate clinical signs or macroscopic evidence on post mortem examination, it cannot be excluded that mild/subclinical gastrointestinal or endocrine disease was present. In acknowledgment of these limitations, this study sought to remain descriptive in nature, to report the identification of immunopositive cells, including their appearance, location and distribution, without seeking to quantify the relative abundance of cells. Immunohistochemistry is a static methodology that identifies the presence or absence of antigens within tissue sections, so inferences about dynamic physiological processes cannot be drawn. Further studies are required to investigate the dynamics of dopamine secretion and the response of different cell types to dopamine receptor binding in horses. However, taken together, this study provides preliminary evidence for the potential role of dopamine to act as a paracrine messenger in the gastrointestinal tract and endocrine pancreas of horses.

## Supporting information

S1 Table Signalment data from six horses included in this study. (PDF)
