# Supplementary material for: Evidence for dopamine production and distribution of dopamine D2 receptors in the equine gastrointestinal mucosa and pancreas
Source: PLoS One. 2024 Feb 27;19(2):e0298660. doi: 10.1371/journal.pone.0298660 (PMC10898723; doi:10.1371/journal.pone.0298660)
Supplement: S1 Table — (PDF) [file pone.0298660.s001.pdf]

S1 Table

Signalment data from six horses included in this study.

Horse	Breed	Age (years)	Reason for euthanasia
1	Thoroughbred	3	Cervical stenotic myelopathy
2	Thoroughbred-cross	2.5	Cervical stenotic myelopathy
3	Thoroughbred	6	Cervical stenotic myelopathy
4	Thoroughbred	11	Large parotid melanomas
5	Thoroughbred	3	Proximal sesamoid bone fracture
6	Standardbred	7	Navicular bone necrosis
